# Subsequent Survival and Loss of Lifetime for Patients With Progression‐Free 24 Months After Treatment in Nasopharyngeal Carcinoma: A Comprehensive Nationwide Population‐Based Analysis

**DOI:** 10.1002/mco2.70143

**Published:** 2025-03-20

**Authors:** Yang Liu, Yaqian Han, Mei Feng, Ye Zhang, Kai Wang, Yuan Qu, Xuesong Chen, Jianghu Zhang, Jingwei Luo, Runye Wu, Ye‐Xiong Li, Xiaodong Huang, Qiuyan Chen, Jingbo Wang, Junlin Yi

**Affiliations:** ^1^ Department of Radiation Oncology National Cancer Center/National Clinical Research Center for Cancer/Cancer Hospital Chinese Academy of Medical Sciences and Peking Union Medical College Beijing China; ^2^ Department of Radiation Oncology Hunan Cancer Hospital and The Affiliated Cancer Hospital of Xiangya School of Medicine Central South University Changsha China; ^3^ Department of Radiation Oncology Sichuan Cancer Hospital and Institute, Sichuan Cancer Center, School of Medicine University of Electronic Science and Technology of China Chengdu China; ^4^ Department of Medical Oncology the Third People's Hospital of Sichuan Chengdu China; ^5^ Department of Nasopharyngeal Carcinoma Sun Yat‐sen University Cancer Centre, State Key Laboratory of Oncology in South China, Collaborative Innovation Centre for Cancer Medicine, Guangdong Key Laboratory of Nasopharyngeal Carcinoma Diagnosis and Therapy Guangzhou China; ^6^ Department of Radiation Oncology National Cancer Center/National Clinical Research Center for Cancer/Hebei Cancer Hospital Chinese Academy of Medical Sciences (CAMS) Langfang China

**Keywords:** early recurrence, loss of lifetime, nasopharyngeal carcinoma, standardized mortality ratio, subsequent survival, surrogate endpoint

## Abstract

Currently, there is little evidence supporting the use of early endpoints to assess primary treatment outcomes in nasopharyngeal carcinoma (NPC). We aim to explore the relationship between 24‐month progression‐free survival (PFS24) and subsequent overall survival (sOS) as well as loss of lifetime (LoL) in NPC patients. sOS is defined as survival from the 24‐month point or progression within 24 months leading to mortality. LoL represents the reduction in life expectancy due to NPC, compared to the general population matched by age, sex, and calendar year. The standardized mortality ratio (SMR) is defined as the ratio of observed mortality to expected mortality. The study included 6315 patients from nonendemic and endemic regions of China. Among them, 5301 patients (83.9%) achieved PFS24, with a 5‐year sOS of 90.2% and an SMR of 1.0. Over a 10‐year period following treatment, the mean LoL was only 0.01 months/year. For most subgroups, patients achieving PFS24 exhibited comparable sOS and LoL with the general population. However, patients failing to achieve PFS24 showed significantly worse outcomes, with 5‐year sOS of 21.9%, SMR of 23.7, and LoL of 6.48 months/year. These notable outcome disparities highlight the importance of PFS24 in NPC risk stratification, patient monitoring, and study design.

## Graphical abstract

Progression‐free survival at 24 months (PFS24) is a valid milestone for risk stratification, patient surveillance, and study design in nasopharyngeal carcinoma (NPC). Patients achieving PFS24 exhibited a comparable subsequent overall survival (sOS) and loss of lifetime (LoL) with the background population. Patients failing to achieve PFS24 had markedly unfavorable outcomes, with significantly reduced 5‐year sOS and higher LoL.

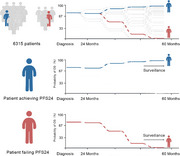

## 1 Introduction

Nasopharyngeal carcinoma (NPC) is the most predominant head and neck cancer in China. A majority of NPC patients present with locally advanced disease, with distant metastases primarily responsible for treatment failure [1, 2]. The overall survival (OS) rates at 5 years for non‐metastatic NPC have reached 80%–90% in the era of comprehensive treatment [1]. Despite implementation of modern treatments, a notable subset of patients still encounter disease progression or recurrence [1, 2]. Consequently, there is a need to improve the overall outcomes in this setting, including early detection of recurrence through close surveillance and the development of more effective treatment modalities through prospective clinical trials.

Progression‐free survival at 24 months (PFS24) is recognized as a key milestone for patient stratification in a variety of malignant hematologic tumors, because subsequent overall survival (sOS) rates after PFS24 are comparable with that of the general (background) population [3, 4, 5, 6]. These novel survival assessment metrics represent meaningful clinical benchmarks to predict the outcomes of cancer patients. However, the implications of PFS24 on NPC remain unclear. The influence of achieving PFS24 on sOS and loss of lifetime (LoL) requires further study. If confirmed, however, PFS24 may become a valid endpoint to assist in the optimization of posttreatment surveillance strategies as well as the acceleration of the clinical trial process by establishing PFS24 as a surrogate endpoint.

To determine the reliability of PFS24 in a real‐world setting, we assessed sOS and LoL stratified by PFS24 using large‐scale individual data of patients with NPC from North, Central, Southwest, and South China.

## 2 Results

### 2.1 Patient Demographics and Survival Outcomes

**FIGURE 1:**
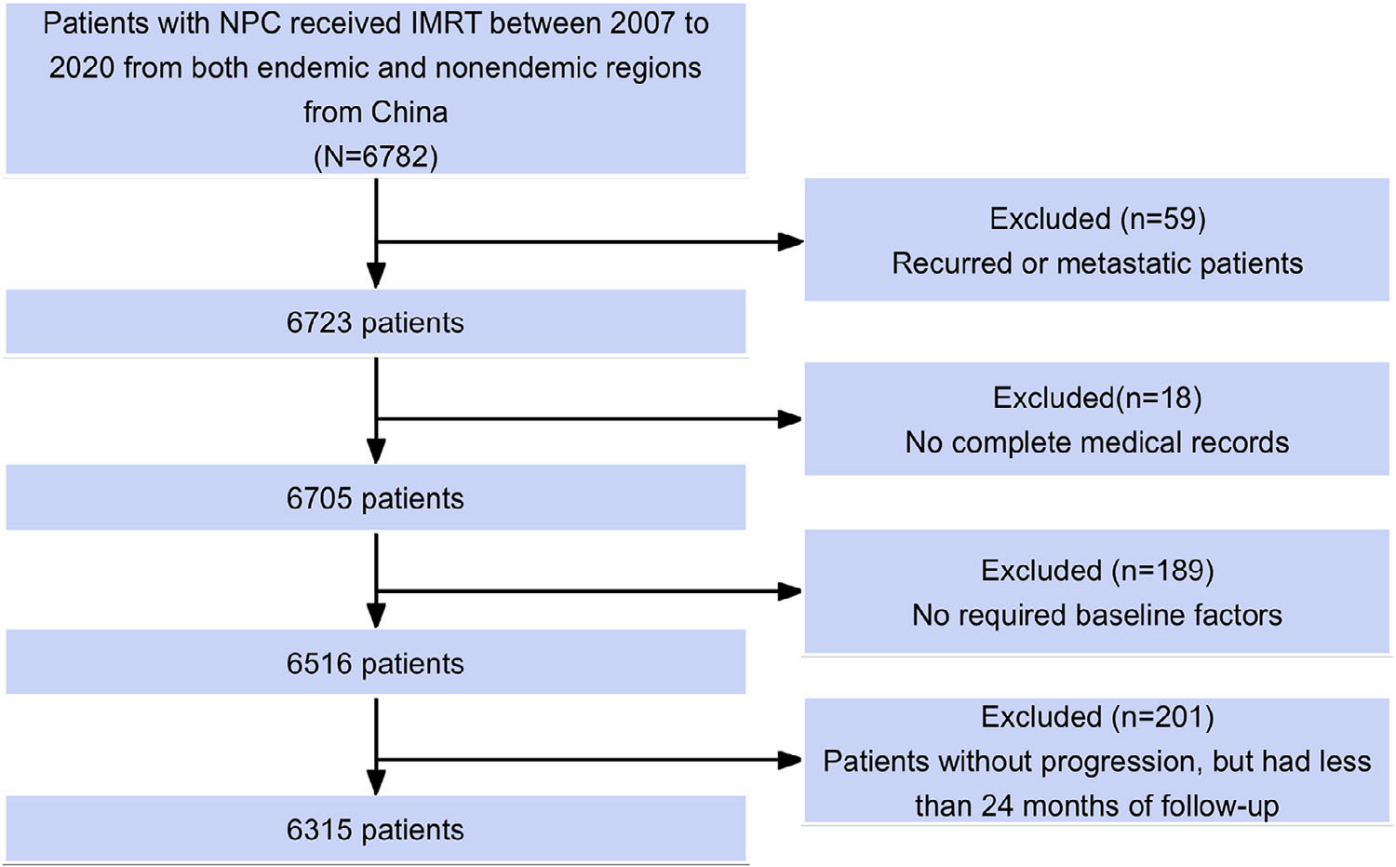
Diagram of the study patient's identification. IMRT, intensity‐modulated radiation therapy; NPC, nasopharyngeal carcinoma.

This comprehensive analysis included 3052 patients from the National Cancer Center cohort (nonendemic region, Beijing) and 3263 patients from the endemic regions, with 1996 patients from Guangzhou, 717 from Sichuan, and 550 from Hunan. Figure 1 provides the comprehensive overview of the process for selecting patients. The ratio of patients from endemic regions to those from nonendemic regions was 1.1:1. Cohort‐specific and aggregated patient features are presented in Table S1. With a median age of 47 years, the cohort had a male‐to‐female ratio of 2.9:1. The majority of patients presented with locally advanced disease (48.2% stage III and 37.4% stage IV). The primary treatment modalities included intensity‐modulated radiation therapy (IMRT) alone in 21.1% of cases, concurrent chemoradiotherapy (CCRT) in 44.9%, and induction chemotherapy (IC) followed by CCRT (IC+CCRT) in 25.2%. A detailed summary is presented in Table 1.

**TABLE 1:** Clinical characteristics and treatment outcomes of 6315 nasopharyngeal carcinoma patients.

Characteristics	No. of patients (%)	5‐Year OS (95% CI)	*p* value	SMR (95% CI)	*p* value
**All patients**	6315 (100)	83.1 (82.0, 84.2)		3.0 (2.9, 3.1)	<0.001^***^
**Sex**			<0.001^***^		
Male	4685 (74.2)	81.4 (80.1, 82.8)		3.0 (2.9, 3.1)	<0.001^***^
Female	1630 (25.8)	86.3 (84.3, 88.3)		2.8 (2.6, 3.0)	<0.001^***^
**Age (years)** ^a^			<0.001^***^		
<48	3181 (50.4)	86.5 (85.1, 87.9)		8.0 (7.8, 8.3)	<0.001^***^
≥48	3134 (49.6)	78.8 (77.1, 80.5)		2.1 (2.0, 2.2)	<0.001^***^
**KPS score**			0.200		
≥80	6076 (96.2)	83.2 (82.1, 84.3)		3.0 (2.9, 3.1)	<0.001^***^
<80	239 (3.8)	79.2 (73.1, 85.8)		3.2 (2.7, 3.7)	<0.001^***^
**Pathology**			<0.001^***^		
WHO II	969 (15.3)	78.3 (75.5, 81.1)		3.4 (3.2, 3.6)	<0.001^***^
WHO III	5158 (81.7)	83.8 (82.6, 85.0)		2.9 (2.8, 3.0)	<0.001^***^
Other	188 (3.0)	79.7 (73.2, 86.8)		2.8 (2.3, 3.3)	<0.001^***^
**AJCC 8th T stage**			<0.001^***^		
T1	816 (12.9)	89.7 (87.3, 92.1)		1.6 (1.4, 1.9)	<0.001^***^
T2	1017 (16.1)	88.4 (86.1, 90.7)		2.1 (1.9, 2.4)	<0.001^***^
T3	2885 (45.7)	86.3 (84.9, 87.8)		2.4 (2.3, 2.6)	<0.001^***^
T4	1597 (25.3)	69.6 (67.0, 72.3)		5.5 (5.4, 5.7)	<0.001^***^
**AJCC 8th N stage**			<0.001^***^		
N0	744 (11.8)	91.0 (88.5, 93.6)		1.2 (1.0, 1.4)	0.300
N1	2189 (34.7)	87.8 (86.1, 89.4)		2.2 (2.0, 2.3)	<0.001^***^
N2	2404 (38.1)	81.1 (79.3, 83.0)		3.5 (3.4, 3.7)	<0.001^***^
N3	978 (15.5)	69.6 (66.3, 73.1)		6.9 (6.6, 7.2)	<0.001^***^
**AJCC 8th clinical stage**			<0.001^***^		
I	188 (3.0)	96.9 (93.9, 100.0)		0.6 (0.3, 1.3)	0.098
II	723 (11.4)	91.7 (89.3, 94.2)		1.5 (1.3, 1.7)	0.006^**^
III	3045 (48.2)	89.6 (88.4, 90.9)		1.8 (1.7, 2.0)	<0.001^***^
IVA	2359 (37.4)	70.3 (68.1, 72.5)		5.7 (5.6, 5.9)	<0.001^***^
**EBV DNA (copies/mL)** ^b^			<0.001^***^		
<2000	4824 (76.4)	83.7 (82.5, 84.9)		2.3 (2.2, 2.5)	<0.001^***^
2000–20,000	942 (14.9)	79.8 (76.4, 83.4)		3.9 (3.6, 4.2)	<0.001^***^
>20000	549 (8.7)	76.2 (71.2, 81.5)		6.5 (6.0, 6.9)	<0.001^***^
**LDH (U/L)** ^c^			<0.001^***^		
<240	5604 (88.8)	83.9 (82.7, 85.0)		2.9 (2.8, 3.0)	<0.001^***^
≥240	711 (11.2)	72.8 (68.7, 77.1)		6.9 (6.5, 7.4)	<0.001^***^
**Treatment modality**			<0.001^***^		
CCRT	2835 (44.9)	83.7 (82.1, 85.3)		3.4 (3.2, 3.5)	<0.001^***^
IMRT	1333 (21.1)	83.8 (81.6, 86.0)		1.7 (1.6, 1.8)	<0.001^***^
IC+CCRT	1594 (25.2)	81.2 (78.6, 84.0)		5.0 (4.7, 5.3)	<0.001^***^
IC+IMRT	234 (3.7)	71.1 (63.9, 79.1)		5.7 (5.1, 6.3)	<0.001^***^
CCRT+AC	135 (2.1)	78.2 (71.0, 86.1)		7.6 (6.6, 8.6)	<0.001^***^
IC+CCRT+AC	145 (2.3)	85.8 (77.7, 94.6)		4.7 (3.7, 6.0)	0.004^**^
IMRT+AC	13 (0.2)	65.8 (43.3, 100.0)		13.7 (10.9, 17.2)	0.026^*^
IC+IMRT+AC	26 (0.4)	96.0 (88.6, 100.0)		1.8 (0.4, 7.5)	0.659

Abbreviations: AC, adjuvant chemotherapy; AJCC, American Joint Committee on Cancer; CCRT, concurrent chemoradiotherapy; CI, confidence interval; EBV DNA, Epstein–Barr virus DNA; IC, induction chemotherapy; IMRT, intensity‐modulated radiation therapy; KPS, Karnofsky Performance Status; LDH, lactate dehydrogenase; OS, overall survival; SMR, standardized mortality ratio.

^a^ Age was dichotomized into two subgroups based on the median value.

^b^ The cutoff of pretreatment cfEBV‐DNA was set at 2000 copies/mL based on previous well‐recognized studies.

^c^ The cutoff values of LDH (240 U/L) have demonstrated powerful prognostic value in previous research.

* *p* < 0.05.

** *p* < 0.01.

*** *p* < 0.001.

**FIGURE 2:**
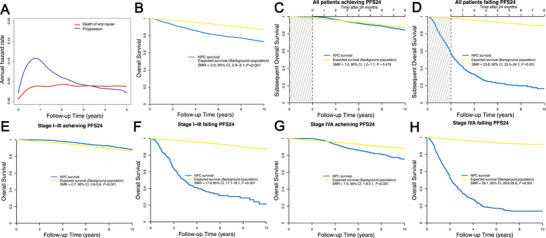
(A) The annual hazard rate trajectory for overall death and progression in the entire cohort. OS of NPC versus the expected OS based on the general population for (B) all patients; (C) all patients achieving PFS24; (D) all patients failing PFS24; (E) stage I–III patients achieving PFS24; (F) stage I–III patients failing PFS24; (G) stage IVA patients achieving PFS24; (H) stage IVA patients failing PFS24. CI, confidence interval; NPC, nasopharyngeal carcinoma; OS, overall survival; PFS24, 24‐month progression‐free survival following initial treatment; SMR, standardized mortality ratio.

The median follow‐up period for survivors was 76 months, of which 1548 patients (24.5%) experienced progression and 1006 patients (15.9%) died. The 5‐year OS and PFS rates across all cohorts were 83.1% and 74.9%, respectively. The OS and PFS curves are presented in Figure S1. Univariate analysis revealed that sex, age, pathology, TNM staging, Epstein–Barr virus (EBV) DNA copies, lactate dehydrogenase (LDH), and treatment modality were significantly correlated with 5‐year OS (Table 1). Figure 2A shows the annual hazard rate trajectory. Following initial treatment, 1014 (65.5%) cases of progression and 440 (43.7%) deaths occurred within 24 months. In concordance, smoothed hazard plots revealed that the peak annual hazard of progression was 10.3% within the initial 24 months and then decreased continuously. Furthermore, after 24 months, the annual hazard of death remained essentially constant at 4.0%.

The 5‐year OS was considerably lower in the entire study cohort (83.1%) compared with that in the matched background population (94.7%). Additionally, the SMR from the initial treatment was 3.0 (Figure 2B). Of note, Kaplan–Meier analysis had an estimated 2‐year PFS rate of 83.8%, which was most similar to the 5‐year OS rate (83.1%) compared with PFS at 12 months (90.3%) and 36 months (79.9%). This suggests that the PFS24 milestone is an important metric for subsequent evaluations.

### 2.2 PFS24 and Subsequent OS

All 6315 patients completed adequate follow‐up for the PFS24 assessment. Of these, 5301 (83.9%) achieved progression‐free status after 24 months. In cases who achieved PFS24, the median OS was not reached (5‐year sOS, 90.2%; Figure 2C). Minimal disparity was observed in the PFS24‐associated actual death rate compared with the expected death rate (SMR 1.0, *p =* 0.478). Table 1 lists the OS and SMR at 5 years for the subgroups. Conversely, for patients experiencing progression within 24 months, the median sOS was limited to 25.0 months. The 5‐year sOS rate post‐progression was 21.9%, with an SMR of 23.8 (*p* < 0.001; Figure 2D).

### 2.3 Exploratory Subgroup Analysis Between PFS24 and Subsequent OS

Substantial variability of the clinical outcomes in the NPC subgroups prompted us to examine subgroup‐specific outcomes contingent upon PFS24 (Table 2). Patients in most subgroups exhibited actual death rates similar to the expected death rates after reaching PFS24.

**TABLE 2:** Subsequent outcomes based on achieving or failing PFS24 in subgroups.

	Outcomes from time of achieving PFS24 (*n* = 5301)					Outcomes from time of failing PFS24 (*n* = 1014)				
Characteristics	No. of patients (%)	5‐Year sOS (95% CI)	*p* value	SMR (95% CI)	*p* value	No. (%)	5‐Year sOS (95% CI)	*p* value	SMR (95% CI)	*p* value
**All patients**	5301 (83.9)	90.2 (89.0, 91.4)		1.0 (1.0, 1.1)	0.478	1014 (16.1)	21.9 (18.9, 25.4)		23.8 (23.5, 24.1)	<0.001^***^
**Sex**			0.090					0.020^*^		
Male	3890 (83.0)	89.8 (88.4, 91.2)		1.0 (0.9, 1.1)	0.482	795 (17.0)	20.1 (16.8, 24.0)		22.8 (22.4, 23.1)	<0.001^***^
Female	1411 (86.6)	91.2 (88.9, 93.5)		1.1 (0.9, 1.3)	0.471	219 (13.4)	29.2 (22.6, 37.6)		28.2 (27.4, 29.1)	<0.001^***^
**Age (years)**			<0.001^***^					<0.001^***^		
<48	2705 (85.0)	93.6 (92.2, 95.0)		2.4 (2.1, 2.7)	<0.001	476 (15.0)	25.3 (20.6, 31.1)		72.4 (71.5, 73.4)	<0.001^***^
≥48	2596 (82.8)	86.5 (84.5, 88.5)		0.8 (0.7, 0.9)	0.005	538 (17.2)	19.1 (15.4, 23.6)		15.9 (15.5, 16.2)	<0.001^***^
**KPS score**			0.900					0.040^*^		
≥80	5108 (84.1)	90.2 (89.0, 91.4)		1.1 (1.0, 1.2)	0.403	968 (15.9)	22.6 (19.5, 26.2)		24.1 (23.8, 24.1)	<0.001^***^
<80	193 (80.8)	92.1 (86.0, 98.6)		0.8 (0.4, 1.5)	0.524	46 (19.2)	7.3 (2.0, 26.8)		18.4 (17.1, 19.9)	<0.001^***^
**Pathology**			0.300					0.100		
WHO II	767 (79.2)	87.2 (84.3, 90.3)		1.2 (1.0, 1.5)	0.136	202 (20.8)	16.4 (11.3, 23.6)		22.3 (21.6, 23.0)	<0.001^***^
WHO III	4388 (85.1)	90.9 (89.6, 92.2)		1.1 (1.0, 1.2)	0.553	770 (14.9)	23.7 (20.1, 28.0)		24.0 (23.6, 24.4)	<0.001^***^
Other	146 (77.7)	93.5 (86.9, 100.0)		0.6 (0.2, 1.3)	0.111	42 (22.3)	26.1 (14.2, 47.9)		22.1 (20.5, 23.9)	<0.001^***^
**AJCC 8th T stage**			<0.001^***^					<0.001^***^		
T1	732 (89.7)	92.3 (89.5, 95.1)		0.8 (0.6, 1.0)	0.082	84 (10.3)	35.2 (25.3, 49.0)		18.0 (16.9, 19.1)	<0.001^***^
T2	885 (87.0)	93.6 (91.2, 96.0)		0.8 (0.6, 1.1)	0.187	132 (13.0)	32.6 (24.0, 44.1)		24.2 (23.2, 25.3)	<0.001^***^
T3	2477 (85.9)	93.3 (91.7, 94.8)		0.7 (0.6, 0.9)	0.001	408 (14.1)	23.1 (18.3, 29.1)		20.6 (20.1, 21.1)	<0.001^***^
T4	1207 (75.6)	80.8 (77.5, 84.1)		2.1 (1.9, 2.3)	<0.001	390 (24.4)	14.2 (10.3, 19.5)		29.0 (28.4, 29.6)	<0.001^***^
**AJCC 8th N stage**			0.090					0.030^*^		
N0	700 (94.1)	91.7 (88.5, 95.1)		0.6 (0.4, 0.9)	0.001	44 (5.9)	14.1 (4.7, 42.2)		16.1 (15.0, 17.3)	<0.001^***^
N1	1952 (89.2)	91.0 (89.1, 93.0)		1.0 (0.8, 1.1)	0.730	237 (10.8)	22.9 (16.8, 31.3)		16.0 (15.5, 16.6)	<0.001^***^
N2	1966 (81.8)	90.3 (88.4, 92.2)		1.2 (1.1, 1.4)	0.064	438 (18.2)	22.6 (18.2, 28.2)		27.1 (26.5, 27.7)	<0.001^***^
N3	683 (69.8)	85.9 (82.0, 90.0)		1.7 (1.5, 2.1)	0.002	295 (30.2)	22.2 (17.1, 28.7)		32.4 (31.7, 33.2)	<0.001^***^
**AJCC 8th clinical stage**			<0.001^***^					<0.001^***^		
I	186 (98.9)	93.0 (86.5, 99.9)		0.5 (0.2, 1.2)	0.043	2 (1.1)	50.0 (12.5, 100.0)		12.5 (7.2, 21.5)	0.527
II	668 (92.4)	92.3 (89.2, 95.4)		0.9 (0.7, 1.2)	0.480	55 (7.6)	31.6 (18.2, 55.0)		15.3 (14.0, 16.6)	<0.001^***^
III	2697 (88.6)	94.3 (93.0, 95.6)		0.6 (0.5, 0.8)	<0.001	348 (11.4)	29.7 (24.2, 36.5)		18.0 (17.5, 18.6)	<0.001^***^
IVA	1750 (74.2)	83.3 (80.8, 86.0)		1.9 (1.8, 2.1)	<0.001	609 (25.8)	17.1 (13.7, 21.3)		29.1 (28.6, 29.6)	<0.001^***^
**EBV DNA (copies/mL)**			0.600					0.200		
<2000	3216 (66.7)	90.5 (89.2, 91.8)		1.0 (0.9, 1.2)	0.505	715 (14.8)	22.0 (18.6, 26.1)		22.2 (21.7, 22.7)	<0.001^***^
2000–20,000	769 (81.6)	88.4 (83.8, 93.2)		1.2 (0.9, 1.5)	0.424	173 (18.4)	25.6 (18.4, 35.7)		17.9 (17.2, 18.7)	<0.001^***^
>20,000	423 (77.0)	90.4 (84.6, 96.5)		1.1 (0.7, 1.8)	0.654	126 (23.0)	12.5 (4.5, 34.5)		36.5 (35.2, 37.8)	<0.001^***^
**LDH (U/L)**			0.600					0.030^*^		
<240	4778 (85.3)	90.4 (89.1, 91.6)		1.0 (1.0, 1.1)	0.600	826 (14.7)	22.9 (19.6, 26.8)		21.7 (21.4, 22.1)	<0.001^***^
≥240	523 (73.6)	88.2 (83.0, 93.8)		1.2 (0.9, 1.6)	0.418	188 (26.4)	19.8 (13.4, 29.2)		36.3 (35.1, 37.6)	<0.001^***^
**Treatment modality**			<0.001^***^					0.200		
CCRT	2422 (85.4)	89.6 (87.8, 91.4)		1.1 (1.0, 1.2)	0.012	413 (14.6)	19.6 (15.4, 25.0)		32.8 (32.2, 33.4)	<0.001^***^
IMRT	1139 (85.4)	91.1 (89.1, 93.2)		1.3 (1.1, 1.4)	0.002	194 (14.6)	22.4 (16.7, 30.0)		12.4 (11.9, 12.9)	<0.001^***^
IC+CCRT	1301 (81.6)	92.5 (90.0, 95.1)		0.7 (0.6, 0.9)	0.237	293 (18.4)	25.9 (19.6, 34.3)		29.9 (29.1, 30.7)	<0.001^***^
IC+IMRT	186 (79.5)	81.6 (73.2, 91.0)		1.2 (0.9, 1.6)	0.009	48 (20.5)	19.7 (10.4, 37.3)		24.0 (22.6, 25.6)	<0.001^***^
CCRT+AC	102 (75.6)	94.6 (88.8, 100.0)		2.4 (1.8, 3.2)	0.948	33 (24.4)	21.3 (10.5, 42.9)		42.7 (40.2, 45.3)	<0.001^***^
IC+CCRT+AC	118 (81.4)	96.7 (94.8, 98.6)		1.0 (0.3, 2.9)	0.733	27 (18.6)	27.4 (19.9, 34.9)		27.2 (24.2, 30.6)	<0.001^***^
IMRT+AC	10 (76.9)	–		1.2 (0.5, 3.4)	0.110	3 (23.1)	–		27.3 (21.0, 35.4)	0.194
IC+IMRT+AC	23 (88.5)	–		10.3 (7.3, 14.5)	<0.001	3 (11.5)	–		83.8 (67.7, 103.7)	0.427

Abbreviations: AC, adjuvant chemotherapy; AJCC, American Joint Committee on Cancer; CCRT, concurrent chemoradiotherapy; CI, confidence interval; EBV DNA, Epstein–Barr virus DNA; IC, induction chemotherapy; IMRT, intensity‐modulated radiation therapy; KPS, Karnofsky Performance Status; LDH, lactate dehydrogenase; PFS, progression‐free survival; SMR, standardized mortality ratio.; sOS, subsequent overall survival.

* *p* < 0.05.

*** *p* < 0.001.

Of note, despite significant the divergence of OS among the subgroups with distinct baseline EBV DNA abundance (Table 1), these differences disappeared after achieving PFS24, with a 5‐year sOS of 90.5% for the <2000 subgroup, 88.4% for the 2000–20,000 subgroup, and 90.4% for >20,000 subgroup (*p =* 0.600; Table 2). Similarly, comparable sOS values were observed between LDH‐stratified groups after achieving PFS24 (90.4% for <240 U/L subgroup, and 88.2% for ≥240 U/L subgroup, *p* = 0.600).

Subsequent analyses examined outcomes related to clinical staging (Table 3). In early‐ to mid‐stage disease, PFS24 was achieved in 98.9% of stage I, 92.4% of stage II, and 88.6% of stage III patients. For patients achieving PFS24, sOS at 5 years was 93.0%, 92.3%, and 94.3% for stage I, II, and III patients, respectively, which is similar to the general population matched by age and sex (92.2%, 94.3%, and 94.6%, Figure 2E; Table 3). The SMRs for stage I, II, and III patients achieving PFS24 were 0.5, 0.9, and 0.6, respectively. Conversely, for patients failing PFS24, the 5‐year sOS was markedly lower at 50.0%, 31.6%, and 29.7% for stages I, II, and III, respectively (Figure 2F), with corresponding SMR values of 12.5, 15.3, and 18.0.

**TABLE 3:** SMR and OS after achieving or failure of PFS at selected time points.

				3 Years from time point			5 Years from time point		
PFS time point	No. of patients	SMR (95% CI)	*p* value	No. at risk	Actual 3‐year OS (%)	Expected 3‐year OS (%)	No. at risk	Actual 5‐year OS (%)	Expected 5‐year OS (%)
**Patients achieving PFS24**									
**All stages (I—IVA)**									
12 months	5710	1.7 (1.6, 1.8)	<0.001^***^	2868	92.7 (92.0, 93.5)	97.0	1693	87.8 (86.7, 88.9)	94.5
24 months	5301	1.0 (1.0, 1.1)	0.478	2169	94.1 (93.2, 94.9)	97.1	1111	90.2 (89.0, 91.4)	94.6
36 months	3818	0.8 (0.7, 0.9)	<0.001^***^	1626	94.9 (94.0, 95.8)	97.0	795	91.7 (90.4, 93.0)	94.6
**Stage I**									
12 months	187	0.5 (0.2, 1.2)	0.042^*^	95	98.6 (96.7, 100.0)	96.7	57	97.5 (94.7, 100.0)	92.6
24 months	186	0.5 (0.2, 1.2)	0.043^*^	75	97.5 (94.6, 100.0)	96.8	30	93.0 (86.5, 99.9)	92.2
36 months	138	0.4 (0.1, 1.3)	0.006^**^	56	98.9 (96.7, 100.0)	95.6	26	90.9 (82.3, 100.0)	92.9
**Stage II**									
12 months	699	1.2 (1.0, 1.4)	0.275	325	95.0 (93.2, 96.9)	96.9	236	92.6 (90.1, 95.2)	94.3
24 months	668	0.9 (0.7, 1.2)	0.480	267	96.0 (94.0, 97.9)	96.9	193	92.3 (89.2, 95.4)	94.3
36 months	482	0.7 (0.5, 1.0)	0.035^*^	227	97.7 (96.1, 99.3)	97.0	158	93.5 (90.4, 96.7)	93.9
**Stage III**									
12 months	2843	1.0 (0.9, 1.2)	0.736	1446	96.0 (95.2, 96.8)	97.1	858	92.1 (90.8, 93.5)	94.5
24 months	2697	0.6 (0.5, 0.8)	<0.001^***^	1129	96.9 (96.0, 97.7)	97.1	558	94.3 (93.0, 95.6)	94.6
36 months	1956	0.5 (0.4, 0.7)	<0.001^***^	832	96.2 (95.1, 97.3)	96.9	381	94.7 (93.2, 96.2)	94.6
**Stage IVA**									
12 months	1981	3.2 (3.1, 3.4)	<0.001^***^	1033	86.9 (85.2, 88.6)	97.1	559	79.5 (77.2, 81.8)	94.8
24 months	1750	1.9 (1.8, 2.1)	<0.001^***^	698	89.0 (87.1, 90.9)	97.2	330	83.3 (80.7, 85.9)	95.1
36 months	1242	1.3 (1.2, 1.5)	0.012^*^	511	91.5 (89.6, 93.4)	95.3	230	86.5 (83.8, 89.4)	95.3
**Patients failing PFS24**									
**All stages (I—IV)**									
12 months	605	32.6 (32.2, 33.1)	<0.001^***^	91	24.3 (20.8, 28.4)	97.0	37	15.7 (12.5, 19.7)	95.1
24 months	1014	23.8 (23.5, 24.1)	<0.001^***^	179	30.5 (27.5, 33.9)	97.2	68	21.9 (18.9, 25.4)	95.2
36 months	1220	19.9 (19.6, 20.1)	<0.001^***^	210	31.4 (28.5, 34.5)	97.1	81	23.3 (20.4, 26.6)	95.0
**Stage I**									
12 months	1	21.7 (14.4, 32.7)	–	–		–	–	–	–
24 months	2	12.5 (7.2, 21.5)	0.527	1	50.0 (12.5, 100.0)	98.6	1	50.0 (12.5, 100.0)	98.6
36 months	4	11.4 (8.2, 15.7)	0.071	1	25.0 (4.6, 100.0)	98.6	1	25.0 (4.6, 100.0)	98.6
**Stage II**									
12 months	24	21.3 (19.3, 23.6)	<0.001^***^	3	41.7 (26.0, 66.9)	97.0	2	13.9 (2.6, 73.7)	93.8
24 months	55	15.3 (14.0, 16.6)	<0.001^***^	10	47.4 (35.6, 63.1)	97.0	5	31.6 (18.2, 55.0)	94.7
36 months	74	11.4 (10.5, 12.4)	<0.001^***^	15	43.6 (32.5, 58.6)	96.2	8	32.8 (21.1, 51.0)	94.3
**Stage III**									
12 months	202	28.1 (27.2, 28.9)	<0.001^***^	41	30.7 (24.4, 38.3)	97.3	22	24.6 (18.6, 32.5)	95.6
24 months	348	18.0 (17.5, 18.6)	<0.001^***^	77	38.2 (32.9, 44.4)	97.1	33	29.7 (24.2, 36.5)	94.8
36 months	416	15.6 (15.2, 16.1)	<0.001^***^	93	41.1 (36.1, 46.9)	97.1	39	32.0 (26.7, 38.4)	94.8
**Stage IVA**									
12 months	378	36.5 (35.9, 37.2)	<0.001^***^	47	20.1 (16.0, 25.1)	96.7	13	10.6 (7.2, 15.5)	94.7
24 months	609	29.1 (28.6, 29.6)	<0.001^***^	90	24.9 (21.3, 29.1)	97.2	30	17.1 (13.7, 21.3)	95.4
36 months	726	24.4 (24.0, 24.8)	<0.001^***^	101	24.8 (21.4, 28.7)	97.1	33	17.6 (14.3, 21.5)	95.3

Abbreviations: CI, confidence interval; OS, overall survival; PFS, progression‐free survival; SMR, standardized mortality ratio.

* *p* < 0.05.

** *p* < 0.01.

*** *p* < 0.001.

For advanced stage IVA disease, however, PFS24 was only achieved in 74.2% of the patients. Patients achieving PFS24 had a 5‐year sOS rate of 83.3% with an SMR of 1.9, which indicates a significantly worse survival compared with the general population (95.1%; Figure 2G; Table 3). For patients failing to achieve PFS24, the 5‐year sOS rate was considerably lower at 17.1% (general population, 95.5%; Figure 2H), with a high SMR of 29.1.

### 2.4 Loss of Residual Lifetime Analysis and Cumulative Mortality

**FIGURE 3:**
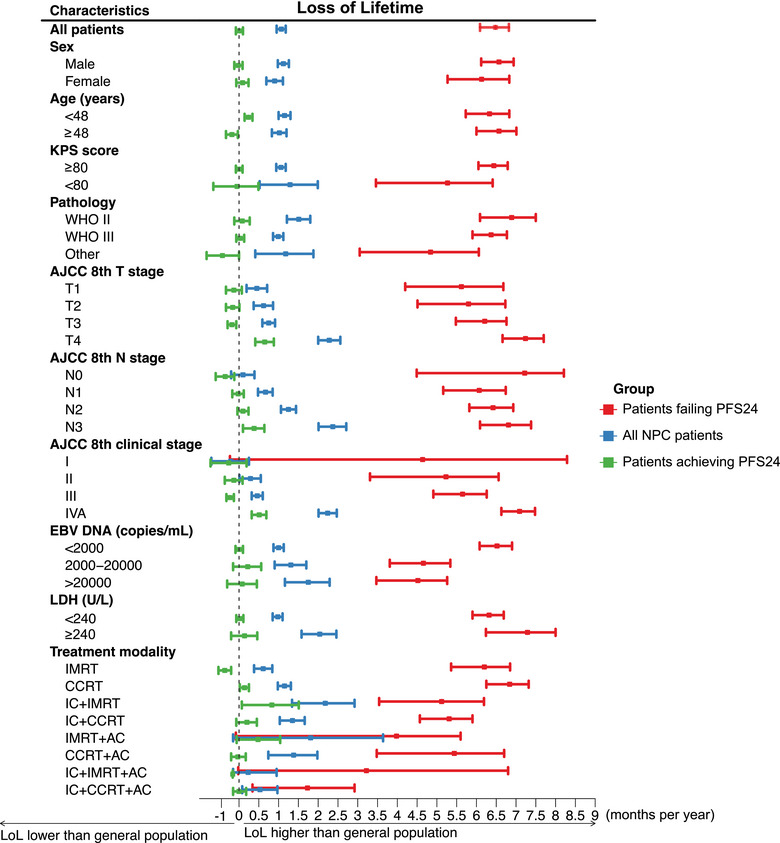
Loss of lifetime in NPC subgroups compared with the general population for all patients, patients achieving PFS24, and patients failing PFS24. AC, adjuvant chemotherapy; AJCC, American Joint Committee on Cancer; CCRT, concurrent chemoradiotherapy; CI, confidence interval; EBV DNA, Epstein–Barr virus DNA; IC, induction chemotherapy; IMRT, intensity‐modulated radiation therapy; KPS, Karnofsky Performance Status; LDH, lactate dehydrogenase; LoL, loss of lifetime; NPC, nasopharyngeal carcinoma; PFS24, 24‐month progression‐free survival following initial treatment.

Throughout the posttreatment phase, NPC patients experienced a reduction in residual lifetime by 1.09 months per year within the initial 10 years, compared to the expected survival for the background population. The detailed LoL outcomes by NPC subgroup are presented in Figure 3 and Table S2. For patients achieving PFS24, the residual lifetime was reduced by 0.01 months/year. In most patients achieving PFS24, the reduction in lifetime was less pronounced and statistically insignificant compared with the background population, even among those with an age ≥48 years, KPS score <80, EBV DNA ≥2000 copies/mL, and an LDH ≥240 U/L (Figure 3). For T4, N3, and stage IVA disease, however, LoL remained significantly high at 0.65, 0.38, and 0.51 months/year, respectively. For patients failing to achieve PFS24, the corresponding LoL estimates were 6.48 months/year (Figure 3).

The 5‐year cumulative incidence of mortality in all patients was 17.3%, whereas a reduction to 6.0% was observed in patients who successfully achieved PFS24 (Figure S2A). Similarly, the cumulative incidence of progression at 5 years was 25.1% in all patients with NPC, which decreased to 10.8% in patients achieving PFS24 (Figure S2B). Multivariate analysis for individuals achieving PFS24 revealed that age >48 years, advanced T and N stage, high EBV DNA copies, and treatment without concurrent chemotherapy were related factors for late relapse (Table S3). Additionally, for individuals achieving PFS24, age >48 years, advanced T stage, and treatment without concurrent chemotherapy were strongly correlated with late mortality (Table S4).

### 2.5 Sensitivity Analysis Comparing PFS24 With Alternative Time Points

**FIGURE 4:**
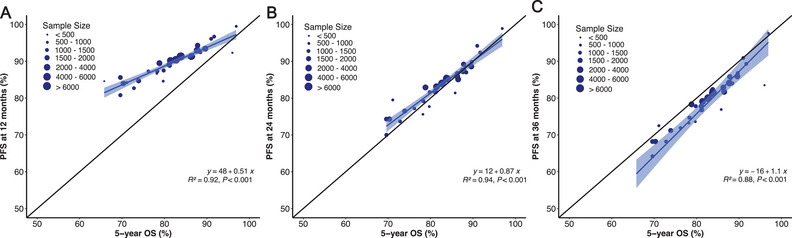
Association of PFS at different time points and 5‐year OS by generating corresponding rates within distinct subgroups. Squared linear correlations of (A) 12‐month progression‐free survival (PFS12), (B) 24‐month progression‐free survival (PFS24), and (C) 36‐month progression‐free survival (PFS36) with 5‐year OS. The squared linear correlation adjusted for measurement error was strong with an *R* ^2^ coefficient of 0.94 between treatment effects on PFS24 and 5‐year OS. OS, overall survival.

In the context of sensitivity analysis, we examined outcomes at the additional time points of PFS12 and PFS36. The squared linear correlation after adjustment for measurement error was strong, with an *R* ^2^ coefficient of 0.94 between the treatment effect of PFS24 and 5‐year OS (Figure 4). The squared linear correlations of PFS12 and PFS36 with 5‐year OS were suboptimal, yielding coefficients *R^2^* of 0.92 and 0.88, respectively.

For patients achieving PFS24, we observed no significant difference in the actual death rate at PFS24 compared with the expected death rate (SMR 1.0, *p* = 0.478); however, the actual death rates for patients achieving PFS12 and PFS36 were significantly different compared with those of the background population (SMR: 1.7 at 12 months and 0.7 at 36 months; Table 3). Furthermore, for patients failing to achieve the selected time point, there was minimal difference in 5‐year sOS rates at 24 (22.0%) and 36 (23.3%) months, suggesting that there was minimal advantage in extending the timeframe beyond 24 months. Consistent outcomes were noted in patients with early‐to‐mid‐stage disease (Table 3); however, for stage IVA patients, despite achieving PFS12, PFS24, or PFS36, the three‐ and 5‐year sOS remained significantly lower than the expected survival rate in the background population, and the SMR was significantly higher (Table 3).

## 3 Discussion

Using extensive multicenter data from both endemic and nonendemic regions, the prognostic value of PFS24 for subsequent survival and LoL was assessed in the context of a real‐world contemporary treatment paradigm for NPC. Irrespective of stage, PFS24 proved to be an important milestone for subsequent outcomes in NPC. Patients exhibiting no progression within the initial 24 months following treatment experienced significantly superior long‐term outcomes compared with those failing to achieve PFS24. Specifically, for stage I–III, patients who achieved PFS24 showed a comparable sOS and LoL compared with a general population matched by age and sex (i.e., these patients were “truly curable”). In this early‐to‐intermediate setting, PFS24 may be considered a reliable marker for disease cure in daily practice and a surrogate endpoint for clinical trials involving NPC patients. In contrast, for stage IVA patients, the overall outcome remained unsatisfactory, despite achieving PFS24, with sOS and LoL significantly inferior to the background population. These observations suggest that PFS24 is a useful milestone for optimizing posttreatment surveillance and incorporating it into the clinical trial process as a surrogate endpoint.

Multiple pretreatment characteristics have been demonstrated as important prognostic factors and have been integrated into risk stratification and treatment modification models [7, 8, 9, 10, 11, 12]. In the present study, most NPC patients stratified by pretreatment factors were initially at high risk or both progression and mortality; however, a markedly attenuated significance of these risk features was observed after achieving PFS24, even in the high‐risks with high EBV DNA copies and LDH. These results suggest that pretreatment risk factors gradually lose prognostic significance during dynamic surveillance.

In the present study, PFS24 status was significantly associated with sOS and LoL in the entire population, beyond prognostic categorization at diagnosis, and may be considered a prognostic indicator. Most individuals who achieved PFS24 had life expectancies comparable with the general population, demonstrating an SMR of 1.0 and a LoL of merely 0.01 months/year. Therefore, for patients who have reached PFS24, caregivers may recommend a lower intensity of surveillance to minimize mental stress as well as medical costs for surveillance workups. However, a subgroup analysis revealed that stage IV was an exception. Even with the most advanced treatment modality, the prognosis for stage IVA patients remained poor [13, 14]. This suggests that despite improved sOS and lower LoL in stage IVA patients who reached PFS24, they remained essentially “incurable,” thus justifying more intensive treatment for this substage of patients.

Shorter intervals for tumor progression are typically concomitant with diminished OS rates in various solid malignancies [15, 16, 17], including NPC [18, 19]. Consistent with previous studies, for patients unable to attain progression‐free status at 24 months, the median OS following relapse was merely 25 months. This highlights the limited success of treatment in patients with early disease deterioration and necessitates more accurate prediction for patients with a potential for early progression, and consequently, more intensive therapy.

Besides the prognostic value of PFS24 on sOS, we also found a robust linear correlation between PFS24 and 5‐year OS, with an *R^2^* coefficient of 0.94. For stage I to III patients without disease progression at 24 months, the sOS and LoL were similar to the age‐ and gender‐adjusted normal background population. These results indicate the potential value of PFS24 as an early endpoint in clinical trial design. Knowledge of early efficacy endpoints is imperative to facilitate the rapid incorporation of optimal therapeutic strategies into clinical practice.

Two previous meta‐analyses based on data from randomized controlled trials (RCTs) examined the correlation between surrogate endpoints, such as EFS and PFS, with OS [20, 21, 22]; however, these two studies provided limited insights into NPC survival, primarily attributable to the frequently restricted follow‐up intervals and the high selectivity observed in the enrolled patient cohorts. Compared with these studies, our study has several notable advantages. First, individual data were derived from patients who received therapeutic interventions outside the framework of clinical trials, providing prognosis estimates in real‐world settings that can be extrapolated to the general population. Second, the datasets were from various multicenter cohorts, including both endemic and nonendemic patients, with reliable data quality and long follow‐up periods. This illustrates the applicability of our findings to both endemic and nonendemic practices. Third, this study used SMR and LoL data matched to the general population, which is suitable for quantifying long‐term survival and does not rely on accurate cause‐of‐death reporting. Collectively, PFS24 is a convincing and effective surrogate for OS, and warrants further assessment of its validity as a study endpoint for prospective settings involving NPC.

There are several limitations to this study. First, its retrospective design introduces potential biases, which is an inherent limitation. Second, the method employed for detecting EBV DNA are prone to considerable variation among laboratories, which could compromise their broader applicability. Third, differences in electronic health record systems, clinician documentation practices, and patient reporting can introduce biases. These considerations necessitate a cautious approach when interpreting our findings and their applicability to other contexts. Consequently, additional validation in varied clinical environments is needed to support the conclusions drawn from this study.

In conclusion, the evaluation of PFS24 enables the stratification of subsequent prognosis in non‐metastatic NPC patients. Most patients achieving PFS24 exhibit favorable subsequent survival and minimal LoL, which are comparable to the general population and represent a curable entity. Nevertheless, stage IVA patients still succumb to significant and persistent disease failure despite achieving PFS24. This represents an incurable setting and warrants indefinite surveillance to timely detect recurrence. Not surprisingly, patients with early disease progression have a markedly unfavorable prognosis and require more effective subsequent line therapy. Our findings suggest that PFS24 is a robust metric for recommending a surveillance strategy and a promising surrogate endpoint for clinical trial design in NPC.

## 4 Materials and Methods

### 4.1 Study Design

In the present study, 6782 patients with non‐metastatic NPC were retrospectively reviewed from the period spanning January 2007 to December 2020. The inclusion criteria encompassed patients treated with IMRT and those with complete baseline characteristics. To ensure analysis reliability, patients without progression but with follow‐up periods of less than 24 months were excluded. Ultimately, the final cohort consisted of 6315 patients with non‐metastatic NPC from four distinct regions of China. Clinical and demographic information were systematically collected. The study protocol was reviewed and approved by the Medical Research Ethics Committee of the National Cancer Center, Chinese Academy of Medical Sciences (Approval No. 23/353‐4095). Given the retrospective nature of the study, which involved the use of anonymized data, the Committee waived the requirement for informed consent from individual participants. All procedures adhered to ethical standards as outlined in the Declaration of Helsinki, and the confidentiality and privacy of all patients were strictly maintained throughout the study.

### 4.2 Treatment and Follow‐up

Each eligible patient received curative IMRT, with doses of 2.0–2.27 Gy per fraction, administered 5 days per week over a treatment duration of 6–7 weeks. The treatment targeted primary tumors and metastatic lymph nodes with doses exceeding 66 Gy, while regions prone to infiltration were given a minimum of 50 Gy. The chemotherapy regimen—including induction, concurrent, and adjuvant phases—was selected at the discretion of the treating clinician, with platinum‐based agents being the most commonly used.

Systematic evaluations were conducted every 3 months during the initial 2 years, transitioning to semi‐annual evaluations for the following 3–5 years, and subsequently transitioning to annual examinations.

### 4.3 Statistical Analysis

Categorical variables were described using frequencies and percentages, and continuous variables were summarized as medians with ranges. Continuous variables were classified using widely accepted cutoff points. Prior research has established that EBV DNA load cutoffs of 2000 and 20,000 copies/mL possess significant prognostic implications [8, 23]. Kaplan–Meier analysis was used to generate survival estimates. The annual hazard rates delineated the temporal trajectories of progression and morality, whereas the Epanechnikov kernel was used for smoothing. Statistical significance was defined as a two‐sided *p* value of 0.05. Data analyses were conducted utilizing SPSS (version 26.0, IBM) and R software (version 4.2.0; http://www.r‐project.org/).

OS represented the time interval from the initiation of treatment to death or last follow‐up. PFS was defined as the time interval from the initiation of treatment to distant metastasis, local‐regional recurrence, or death from any cause. PFS24 was defined as the status of survival without progression 24 months after initial treatment. The sOS is defined as survival from the 24‐month point or progression within 24 months leading to mortality. A general population was derived from Chinese life tables and matched with the study cohort by age, gender, and calendar year using a conditional approach. LoL represented the reduction in life expectancy due to NPC, compared to the general population, such as cancer. The 10‐year LoL was determined by calculating the area under the survival curves between NPC patients and the general population from treatment initiation to a 10‐year follow‐up period. The SMR was defined as the ratio of observed mortality to expected mortality. Expected survival was estimated using a conditional method, with the “survexp” function in R software.

For the sensitivity analysis, we investigated the relationship between the distributions of multipoint PFS rates and 5‐year OS rates through linear regression analysis conducted in R software. This analysis was performed on subgroups defined by predefined, widely recognized prognostic factors. We assessed the correlation between PFS rates at additional landmark time points—namely at 12, 24, and 36 months—and the 5‐year OS rates. The squared linear correlation coefficient (*R^2^*) was calculated to evaluate the association between PFS rates at various time points and 5‐year OS, producing corresponding values for each subgroup. An *R^2^* value approaching 1 indicated a stronger correlation. Based on prior studies, *R^2^* values were categorized as excellent (*R^2^* > 0.9), very good (*R^2^* > 0.75), good (*R^2^* > 0.5), moderate (*R^2^* > 0.25), and poor otherwise.

## Author Contributions

**Yang Liu**: conceptualization, methodology, formal analysis, investigation, writing –original draft, visualization. **Yaqian Han**: data curation, investigation. **Mei Feng**: data curation, investigation. **Ye Zhang**: data curation, investigation. **Kai Wang**: data curation, investigation. **Yuan Qu**: data curation, investigation. **Xuesong Chen**: data curation, investigation. **Jianghu Zhang**: data curation, investigation. **Jingwei Luo**: data curation, investigation. **Runye Wu**: data curation, investigation. **Ye‐Xiong Li**: data curation, investigation. **Xiaodong Huang**: data curation, investigation. **Qiuyan Chen**: data curation, investigation. **Jingbo Wang**: funding acquisition, conceptualization, methodology, formal analysis, investigation, writing – original draft, visualization. **Junlin Yi**: funding acquisition, project administration, resources, writing – review and editing, conceptualization. All authors have read and approved the final manuscript.

## Ethics Statement

The study protocol was reviewed and approved by the Medical Research Ethics Committee of the National Cancer Center, Chinese Academy of Medical Sciences (Approval No. 23/353‐4095). Given the retrospective nature of the study, which involved the use of anonymized data, the Committee waived the requirement for informed consent from individual participants. All procedures adhered to ethical standards as outlined in the Declaration of Helsinki, and the confidentiality and privacy of all patients were strictly maintained throughout the study.

## Conflicts of Interest

The authors declare no conflicts of interest.

## Supporting information



Supporting Information

## Data Availability

The datasets used or analyzed in this study are available upon request from the corresponding author.

## References

[1] K. H. Au , R. K. C. Ngan , A. W. Y. Ng , et al., “Treatment Outcomes of Nasopharyngeal Carcinoma in Modern Era After Intensity Modulated Radiotherapy (IMRT) in Hong Kong: A Report of 3328 Patients (HKNPCSG 1301 Study),” Oral Oncology 77 (2018): 16–21. 29362121 10.1016/j.oraloncology.2017.12.004

[2] W. T. Ng , J. Corry , J. A. Langendijk , et al., “Current Management of Stage IV Nasopharyngeal Carcinoma Without Distant Metastasis,” Cancer Treatment Reviews 85 (2020): 101995. 32113080 10.1016/j.ctrv.2020.101995

[3] L. H. Jakobsen , M. Bogsted , P. N. Brown , et al., “Minimal Loss of Lifetime for Patients with Diffuse Large B‐Cell Lymphoma in Remission and Event Free 24 Months After Treatment: A Danish Population‐Based Study,” Journal of Clinical Oncology 35, no. 7 (2017): 778–784. 28095160 10.1200/JCO.2016.70.0765

[4] M. J. Maurer , T. M. Habermann , Q. Shi , et al., “Progression‐Free Survival at 24 Months (PFS24) and Subsequent Outcome for Patients With Diffuse Large B‐Cell Lymphoma (DLBCL) Enrolled on Randomized Clinical Trials,” Annals of Oncology 29, no. 8 (2018): 1822–1827. 29897404 10.1093/annonc/mdy203 PMC6096732

[5] M. J. Maurer , F. Ellin , L. Srour , et al., “International Assessment of Event‐Free Survival at 24 Months and Subsequent Survival in Peripheral T‐Cell Lymphoma,” Journal of Clinical Oncology 35, no. 36 (2017): 4019–4026. 29072976 10.1200/JCO.2017.73.8195 PMC5736237

[6] Y. Yang , Y. Wang , X. Liu , et al., “Progression‐Free Survival at 24 Months and Subsequent Survival of Patients With Extranodal NK/T‐Cell Lymphoma: A China Lymphoma Collaborative Group (CLCG) Study,” Leukemia 35, no. 6 (2021): 1671–1682. 32943751 10.1038/s41375-020-01042-y PMC8179849

[7] L. Q. Tang , C. F. Li , J. Li , et al., “Establishment and Validation of Prognostic Nomograms for Endemic Nasopharyngeal Carcinoma,” JNCI: Journal of the National Cancer Institute 108, no. 1 (2016): djv291. 10.1093/jnci/djv291 26467665

[8] R. Guo , L. L. Tang , Y. P. Mao , et al., “Proposed Modifications and Incorporation of Plasma Epstein‐Barr Virus DNA Improve the TNM Staging System for Epstein‐Barr Virus‐Related Nasopharyngeal Carcinoma,” Cancer 125, no. 1 (2019): 79–89. 30351466 10.1002/cncr.31741

[9] V. H. Lee , D. L. Kwong , T. W. Leung , et al., “The Addition of Pretreatment Plasma Epstein‐Barr Virus DNA Into the Eighth Edition of Nasopharyngeal Cancer TNM Stage Classification,” International Journal of Cancer 144, no. 7 (2019): 1713–1722. 30192385 10.1002/ijc.31856

[10] J. J. Pan , W. T. Ng , J. F. Zong , et al., “Prognostic Nomogram for Refining the Prognostication of the Proposed 8th Edition of the AJCC/UICC Staging System for Nasopharyngeal Cancer in the Era of Intensity‐Modulated Radiotherapy,” Cancer 122, no. 21 (2016): 3307–3315. 27434142 10.1002/cncr.30198 PMC5524130

[11] W. Liang , G. Shen , Y. Zhang , et al., “Development and Validation of a Nomogram for Predicting the Survival of Patients With Non‐Metastatic Nasopharyngeal Carcinoma After Curative Treatment,” Chinese Journal of Cancer 35, no. 1 (2016): 98. 27887636 10.1186/s40880-016-0160-9 PMC5124222

[12] C. Xu , Y. P. Chen , X. Liu , et al., “Establishing and Applying Nomograms Based on the 8th Edition of the UICC/AJCC Staging System to Select Patients With Nasopharyngeal Carcinoma Who Benefit From Induction Chemotherapy Plus Concurrent Chemoradiotherapy,” Oral Oncology 69 (2017): 99–107. 28559028 10.1016/j.oraloncology.2017.04.015

[13] L. L. Tang , Y. P. Chen , Y. P. Mao , et al., “Validation of the 8th Edition of the UICC/AJCC Staging System for Nasopharyngeal Carcinoma from Endemic Areas in the Intensity‐Modulated Radiotherapy Era,” Journal of the National Comprehensive Cancer Network: JNCCN 15, no. 7 (2017): 913–919. 28687579 10.6004/jnccn.2017.0121

[14] J. Wang , X. Huang , S. Sun , et al., “Stage‐Dependent Conditional Survival and Failure Hazard of Non‐metastatic Nasopharyngeal Carcinoma After Intensity‐Modulated Radiation Therapy: Clinical Implications for Treatment Strategies and Surveillance,” Cancer Medicine 10, no. 11 (2021): 3613–3621. 33960136 10.1002/cam4.3917 PMC8178506

[15] N. Portolani , A. Coniglio , S. Ghidoni , et al., “Early and Late Recurrence After Liver Resection for Hepatocellular Carcinoma: Prognostic and Therapeutic Implications,” Annals of Surgery 243, no. 2 (2006): 229–235. 16432356 10.1097/01.sla.0000197706.21803.a1 PMC1448919

[16] K. Imai , M. A. Allard , C. C. Benitez , et al., “Early Recurrence After Hepatectomy for Colorectal Liver Metastases: What Optimal Definition and What Predictive Factors?,” Oncologist 21, no. 7 (2016): 887–894. 27125753 10.1634/theoncologist.2015-0468 PMC4943389

[17] X. F. Zhang , E. W. Beal , J. Chakedis , et al., “Defining Early Recurrence of Hilar Cholangiocarcinoma After Curative‐Intent Surgery: A Multi‐Institutional Study From the US Extrahepatic Biliary Malignancy Consortium,” World Journal of Surgery 42, no. 9 (2018): 2919–2929. 29404753 10.1007/s00268-018-4530-0

[18] X. L. Yang , L. L. Zhang , J. Kou , et al., “Cisplatin‐Based Concurrent Chemoradiotherapy Improved the Survival of Locoregionally Advanced Nasopharyngeal Carcinoma After Induction Chemotherapy by Reducing Early Treatment Failure,” BMC Cancer 22, no. 1 (2022): 1230. 36443685 10.1186/s12885-022-10237-8 PMC9706941

[19] L. L. Zhang , W. H. Zheng , W. J. Zhu , et al., “Prognostic Models for Early and Late Tumor Progression Prediction in Nasopharyngeal Carcinoma: An Analysis of 8292 Endemic Cases,” Cancer Medicine 12, no. 5 (2023): 5384–5396. 36301691 10.1002/cam4.5361 PMC10028159

[20] Y. P. Chen , Y. Sun , L. Chen , et al., “Surrogate Endpoints for Overall Survival in Combined Chemotherapy and Radiotherapy Trials in Nasopharyngeal Carcinoma: Meta‐Analysis of Randomised Controlled Trials,” Radiotherapy and Oncology 116, no. 2 (2015): 157–166. 26243677 10.1016/j.radonc.2015.07.030

[21] F. Rotolo , J. P. Pignon , J. Bourhis , et al., “Surrogate End Points for Overall Survival in Loco‐Regionally Advanced Nasopharyngeal Carcinoma: An Individual Patient Data Meta‐Analysis,” JNCI: Journal of the National Cancer Institute 109, no. 4 (2017): djw239. 27927756 10.1093/jnci/djw239 PMC6059121

[22] S. Zhou , C. Chen , S. R. Liu , et al., “Surrogate Endpoints Shortening the Therapeutic Evaluation Duration for Different Subgroups of Patients With Nasopharyngeal Carcinoma Receiving Intensity‐Modulated Radiotherapy: A Retrospective Analysis of 830 Patients Stratified by the 8th Edition of the UICC/AJCC Staging System and Plasma Epstein‐Barr Viral,” Journal of Cancer 9, no. 18 (2018): 3352–3360. 30271496 10.7150/jca.25530 PMC6160691

[23] A. W. M. Lee , V. H. F. Lee , W. T. Ng , et al., “A Systematic Review and Recommendations on the Use of Plasma EBV DNA for Nasopharyngeal Carcinoma,” European Journal of Cancer 153 (2021): 109–122. 34153713 10.1016/j.ejca.2021.05.022

