# Supplementary material for: Subsequent Survival and Loss of Lifetime for Patients With Progression‐Free 24 Months After Treatment in Nasopharyngeal Carcinoma: A Comprehensive Nationwide Population‐Based Analysis
Source: MedComm (2020). 2025 Mar 20;6(4):e70143. doi: 10.1002/mco2.70143 (PMC11923380; doi:10.1002/mco2.70143)
Supplement: Supplementary file 1 — Supporting Information [file MCO2-6-e70143-s001.pdf]

**Table S1. Patient demographic and clinical characteristics by cohort.**

| Characteristics | Non-endemic region (*n*=3052) | Endemic region (*n*=3263) | | | Overall (*N*=6315) | *P* value |
|---|---|---|---|---|---|---|
| | NCC cohort, % (*n*=3052) | Guangzhou cohort, % (*n*=1996) | Sichuan cohort, % (*n*=717) | Hunan cohort, % (*n*=550) | | |
| **Sex** | | | | | | 0.482 |
| Male | 2256 (73.9) | 1500 (75.2) | 533 (74.3) | 396 (72.0) | 4685 (74.2) | |
| Female | 796 (26.1) | 496 (24.8) | 184 (25.7) | 154 (28.0) | 1630 (25.8) | |
| **Age (years)** | | | | | | <0.001 |
| <48 | 1487 (48.7) | 1141 (57.2) | 325 (45.3) | 228 (41.5) | 3181 (50.4) | |
| ≥48 | 1565 (51.3) | 855 (42.8) | 392 (54.7) | 322 (58.5) | 3134 (49.6) | |
| **KPS score** | | | | | | <0.001 |
| ≥80 | 2966 (97.2) | 1862 (93.3) | 700 (97.6) | 548 (99.6) | 6076 (96.2) | |
| < 80 | 86 (2.8) | 134 (6.7) | 17 (2.4) | 2 (0.4) | 239 (3.8) | |
| **Pathology** | | | | | | <0.001 |
| WHO II | 677 (22.2) | 72 (3.6) | 147 (20.5) | 73 (13.3) | 969 (15.3) | |
| WHO III | 2214 (72.5) | 1924 (96.4) | 543 (75.7) | 477 (86.7) | 5158 (81.7) | |
| Other | 161 (5.3) | 0 (0) | 27 (3.8) | 0 (0) | 188 (3.0) | |
| **AJCC 8th T stage** | | | | | | <0.001 |
| T1 | 428 (14.0) | 228 (11.4) | 74 (10.3) | 86 (15.6) | 816 (12.9) | |
| T2 | 468 (15.3) | 335 (16.8) | 98 (13.7) | 116 (21.1) | 1017 (16.1) | |
| T3 | 1306 (42.8) | 1003 (50.3) | 352 (49.1) | 224 (40.7) | 2885 (45.7) | |
| T4 | 850 (27.9) | 430 (21.5) | 193 (26.9) | 124 (22.5) | 1597 (25.3) | |
| **AJCC 8th N stage** | | | | | | <0.001 |
| N0 | 268 (8.8) | 399 (20.0) | 58 (8.1) | 19 (3.5) | 744 (11.8) | |
| N1 | 990 (32.4) | 741 (37.1) | 243 (33.9) | 215 (39.1) | 2189 (34.7) | |
| N2 | 1230 (40.3) | 688 (34.5) | 288 (40.2) | 198 (36.0) | 2404 (38.1) | |
| N3 | 564 (18.5) | 168 (8.4) | 128 (17.9) | 118 (21.5) | 978 (15.5) | |
| **AJCC 8th clinical stage** | | | | | | <0.001 |
| I | 57 (1.9) | 119 (6.0) | 10 (1.4) | 2 (0.4) | 188 (3.0) | |
| II | 336 (11.0) | 226 (11.3) | 64 (8.9) | 97 (17.6) | 723 (11.4) | |
| III | 1374 (45.0) | 1086 (54.4) | 351 (49.0) | 234 (42.5) | 3045 (48.2) | |
| IVA | 1285 (42.1) | 565 (28.3) | 292 (40.7) | 217 (39.5) | 2359 (37.4) | |
| **EBV DNA (copies/mL)** | | | | | | <0.001 |
| <2000 | 2766 (90.6) | 992 (49.7) | 623 (86.9) | 443 (80.5) | 4824 (76.4) | |
| 2000-20000 | 229 (7.5) | 547 (27.4) | 85 (11.9) | 81 (14.7) | 942 (14.9) | |
| >20000 | 57 (1.9) | 457 (22.9) | 9 (1.3) | 26 (4.7) | 549 (8.7) | |
| **LDH (U/L)** | | | | | | <0.001 |
| <240 | 2853 (93.5) | 1539 (77.1) | 688 (96.0) | 524 (95.3) | 5604 (88.7) | |
| ≥240 | 199 (6.5) | 457 (22.9) | 29 (4.0) | 26 (4.7) | 711 (11.3) | |
| **Treatment modality** | | | | | | <0.001 |
| CCRT | 1476 (48.4) | 1084 (54.3) | 263 (36.7) | 12 (2.2) | 2835 (44.9) | |
| IMRT | 845 (27.7) | 305 (15.3) | 174 (24.3) | 9 (1.6) | 1333 (21.1) | |
| IC+CCRT | 516 (16.9) | 552 (27.7) | 201 (28.0) | 325 (59.1) | 1594 (25.2) | |
| IC+IMRT | 135 (4.4) | 0 (0) | 35 (4.9) | 64 (11.6) | 234 (3.7) | |
| CCRT+AC | 60 (2.0) | 55 (2.8) | 20 (2.8) | 0 (0) | 135 (2.1) | |
| IC+CCRT+AC | 8 (0.3) | 0 (0) | 18 (2.5) | 119 (21.6) | 145 (2.3) | |
| IMRT+AC | 8 (0.3) | 0 (0) | 5 (0.7) | 0 (0) | 13 (0.2) | |

| | | | | | |
|---|---|---|---|---|---|
| IC+IMRT+AC | 4 (0.1) | 0 (0) | 1 (0.1) | 21 (3.8) | 26 (0.4) |

Abbreviations: AC, adjuvant chemotherapy; AJCC, American Joint Committee on Cancer; CCRT, concurrent chemoradiotherapy; EBV DNA, Epstein-Barr virus DNA; IC, induction chemotherapy; IMRT, intensity-modulated radiation therapy; KPS, Karnofsky Performance Status; LDH, lactate dehydrogenase; NCC, national cancer center; OS, overall survival; SMR, standardized mortality ratio.

**Table S2. Loss of lifetime for patients in the whole cohort: patients achieving PFS24 and patients not achieving PFS24.**

| Characteristics | All patients | Patients achieving PFS24 (95% CI) | Patients not achieving PFS24 (95% CI) |
|---|---|---|---|
| **All patients** | 1.09 (0.97, 1.20) | 0.01 (-0.08, 0.10) | 6.48 (6.09, 6.82) |
| **Sex** | | | |
| Male | 1.12 (0.98, 1.26) | -0.02 (-0.12, 0.09) | 6.57 (6.12, 6.94) |
| Female | 0.90 (0.69, 1.11) | 0.09 (-0.07, 0.24) | 6.13 (5.27, 6.83) |
| **Age (years)** | | | |
| <48 | 1.15 (1.00, 1.30) | 0.24 (-0.14, 0.34) | 6.33 (5.73, 6.83) |
| ≥48 | 1.02 (0.83, 1.20) | -0.18 (-0.33, 0.03) | 6.57 (6.00, 7.01) |
| **KPS score** | | | |
| ≥80 | 1.06 (0.94, 1.18) | 0.01 (-0.08, 0.09) | 6.44 (6.05, 6.79) |
| < 80 | 1.29 (0.52, 1.99) | -0.05 (-0.65, 0.49) | 5.27 (3.46, 6.41) |
| **Pathology** | | | |
| WHO II | 1.51 (1.21, 1.80) | 0.08 (-0.12, 0.27) | 6.89 (6.09, 7.50) |
| WHO III | 0.99 (0.86, 1.12) | 0.03 (-0.07, 0.13) | 6.37 (5.90, 6.77) |
| Other | 1.18 (0.41, 1.88) | -0.42 (-0.82, 0) | 4.84 (3.05, 6.06) |
| **AJCC 8th T stage** | | | |
| 1 | 0.45 (0.19, 0.71) | -0.13 (-0.33, 0) | 5.62 (4.20, 6.68) |
| 2 | 0.62 (0.37, 0.86) | -0.16 (-0.33, 0) | 5.80 (4.51, 6.73) |
| 3 | 0.75 (0.59, 0.91) | -0.18 (-0.29, 0) | 6.21 (5.48, 6.76) |
| 4 | 2.28 (2.00, 2.56) | 0.65 (0.41, 0.88) | 7.24 (6.66, 7.70) |
| **AJCC 8th N stage** | | | |
| 0 | 0.10 (-0.20, 0.39) | -0.35 (-0.59, -0.12) | 7.22 (4.49, 8.21) |
| 1 | 0.67 (0.48, 0.85) | -0.02 (-0.17, 0.12) | 6.07 (5.16, 6.74) |
| 2 | 1.25 (1.06, 1.44) | 0.10 (-0.04, 0.24) | 6.42 (5.82, 6.93) |
| 3 | 2.37 (2.01, 2.71) | 0.38 (0.10, 0.64) | 6.81 (6.09, 7.38) |
| **AJCC 8th clinical stage** | | | |
| I | -0.37 (-0.74, 0.01) | -0.26 (-0.72, 0.19) | 4.64 (-0.23, 8.29) |
| II | 0.29 (0.01, 0.55) | -0.13 (-0.36, 0.09) | 5.23 (3.31, 6.56) |
| III | 0.46 (0.32, 0.60) | -0.23 (-0.32, -0.13) | 5.65 (4.91, 6.26) |
| IVA | 2.24 (2.01, 2.47) | 0.51 (0.32, 0.69) | 7.09 (6.63, 7.48) |
| **EBV DNA (copies/mL)** | | | |
| <2000 | 1.00 (0.87, 1.13) | 0.00 (-0.09, 0.10) | 6.52 (6.08, 6.90) |
| 2000-20000 | 1.31 (0.90, 1.70) | 0.22 (-0.15, 0.56) | 4.66 (3.81, 5.34) |
| >20000 | 1.75 (1.16, 2.29) | 0.08 (-0.30, 0.45) | 4.52 (3.47, 5.26) |
| **LDH (U/L)** | | | |
| <240 | 0.98 (0.85, 1.10) | 0.02 (-0.07, 0.11) | 6.32 (5.90, 6.69) |
| ≥240 | 2.04 (1.58, 2.46) | 0.14 (-0.20, 0.46) | 7.29 (6.24, 8.00) |
| **Treatment modality** | | | |
| IMRT | 0.61 (0.38, 0.84) | -0.36 (-0.52, -0.20) | 6.20 (5.36, 6.85) |
| CCRT | 1.15 (0.98, 1.31) | 0.14 (-0.02, 0.25) | 6.84 (6.25, 7.32) |

| | | | |
|---|---|---|---|
| IC+IMRT | 2.18 (1.34, 2.92) | 0.83 (-0.07, 1.51) | 5.12 (3.54, 6.19) |
| IC+CCRT | 1.35 (1.03, 1.66) | 0.20 (-0.07, 0.45) | 5.31 (4.57, 5.90) |
| IMRT+AC | 1.81 (-0.15, 3.64) | 0.48 (-0.06, 1.04) | 3.98 (-0.08, 5.60) |
| CCRT+AC | 1.38 (0.74, 1.98) | -0.03 (-0.20, 0.17) | 5.44 (3.48, 6.70) |
| IC+IMRT+AC | 0.23 (-0.15, 0.95) | -0.16 (-0.16, -0.16) | 3.22 (-0.02, 6.80) |
| IC+CCRT+AC | 0.53 (0.08, 0.97) | 0.00 (-0.15, 0.18) | 1.73 (0.34, 2.92) |

**Abbreviations:** AC, adjuvant chemotherapy; AJCC, American Joint Committee on Cancer; CCRT, concurrent chemoradiotherapy; CI, confidence interval; EBV DNA, Epstein-Barr virus DNA; IC, induction chemotherapy; IMRT, intensity-modulated radiation therapy; KPS, Karnofsky Performance Status; LDH, lactate dehydrogenase; PFS24, progression-free status at 24 months post initial treatment.

**Table S3. Univariable and multivariable analysis of PFS in patients achieving PFS24 (*n* = 5304).**

| Characteristics | Univariable | | | Multivariable | | |
|---|---|---|---|---|---|---|
| | HR | 95% CI | *P* value | HR | 95% CI | *P* value |
| **Sex** | | | | / | / | / |
| Male | Ref | | | | | |
| Female | 0.89 | 0.73 - 1.08 | 0.238 | | | |
| **Age (years)** | | | | | | |
| <48 | Ref | | | Ref | | |
| ≥48 | 1.58 | 1.33 - 1.88 | <0.001 | 1.66 | 1.40-1.98 | <0.001 |
| **Pathology** | | | 0.066 | / | / | / |
| WHO II | Ref | | | | | |
| WHO III | 0.79 | 0.64 - 0.97 | 0.023 | | | |
| Other | 0.72 | 0.40 - 1.30 | 0.278 | | | |
| **AJCC 8th T stage** | | | <0.001 | | | <0.001 |
| 1 | Ref | | | Ref | | |
| 2 | 1.02 | 0.73 - 1.43 | 0.917 | 1.03 | 0.74-1.45 | 0.853 |
| 3 | 1.05 | 0.79 - 1.39 | 0.747 | 1.09 | 0.81-1.46 | 0.575 |
| 4 | 2.33 | 1.76 - 3.09 | <0.001 | 2.45 | 1.82-3.29 | <0.001 |
| **AJCC 8th N stage** | | | <0.001 | | | <0.001 |
| 0 | Ref | | | Ref | | |
| 1 | 1.25 | 0.91 - 1.72 | 0.164 | 1.31 | 0.95-1.80 | 0.098 |
| 2 | 1.46 | 1.07 - 2.00 | 0.016 | 1.61 | 1.17-2.22 | 0.003 |
| 3 | 2.13 | 1.51 - 3.00 | <0.001 | 2.37 | 1.63-3.43 | <0.001 |
| **EBV DNA (copies/mL)** | | | 0.007 | | | 0.041 |
| <2000 | Ref | | | Ref | | |
| 2000-20000 | **1.10** | 0.84 - 1.45 | 0.492 | 1.02 | 0.77-1.35 | 0.560 |
| >20000 | 1.67 | 1.21 - 2.29 | 0.002 | 1.70 | 1.04-2.79 | 0.034 |
| **LDH (U/L)** | | | | | | |
| <240 | Ref | | | Ref | | |
| ≥240 | 1.35 | 1.02 - 1.80 | 0.037 | 0.87 | 0.56-1.35 | 0.519 |
| **Treatment modalities** | | | <0.001 | | | <0.001 |
| CCRT | Ref | | | Ref | | |
| IMRT | 1.06 | 0.86 - 1.30 | 0.603 | 1.34 | 1.07-1.67 | 0.011 |
| IC+CCRT | 1.31 | 1.04 - 1.65 | 0.022 | 1.04 | 0.81-1.33 | 0.757 |

| | | | | | | |
|---|---|---|---|---|---|---|
| IC+IMRT | 2.17 | 1.47 - 3.21 | <0.001 | 1.75 | 1.17-2.62 | 0.006 |
| CCRT+AC | 0.98 | 0.50 - 1.91 | 0.957 | 0.80 | 0.41-1.56 | 0.511 |
| IC+CCRT+AC | 0.90 | 0.37 - 2.18 | 0.810 | 0.73 | 0.30-1.78 | 0.483 |
| IMRT+AC | 7.05 | 2.90 - 17.10 | <0.001 | 6.74 | 2.76-16.48 | <0.001 |
| IC+IMRT+AC | 0.94 | 0.13 - 6.69 | 0.948 | 1.73 | 0.43-7.01 | 0.441 |

Abbreviations: AC, adjuvant chemotherapy; AJCC, American Joint Committee on Cancer; CCRT, concurrent chemoradiotherapy; CI, confidence interval; EBV DNA, Epstein-Barr Virus DNA; IC, induction chemotherapy; IMRT, intensity-modulated radiation therapy; LDH, lactate dehydrogenase; OS, overall survival; Ref, reference; SMR, standardized mortality ratio.

**Table S4. Univariable and multivariable analysis of sOS in patients achieving PFS24 (*n* = 5304).**

| Characteristics | Univariable | | | Multivariable | | |
|---|---|---|---|---|---|---|
| | HR | 95% CI | *P* value | HR | 95% CI | *P* value |
| **Sex** | | | | / | / | / |
| Male | Ref | | | | | |
| Female | 0.80 | 0.62 - 1.03 | 0.087 | | | |
| **Age (years)** | | | | | | |
| <48 | Ref | | | Ref | | |
| ≥48 | 2.35 | 1.86 - 2.96 | <0.001 | 2.41 | 1.90 - 3.04 | <0.001 |
| **Pathology** | | | 0.260 | / | / | / |
| WHO II | Ref | | | | | |
| WHO III | 0.84 | 0.64 - 1.09 | 0.178 | | | |
| Other | 0.59 | 0.26 - 1.36 | 0.216 | | | |
| **AJCC 8th T stage** | | | <0.001 | | | <0.001 |
| 1 | Ref | | | Ref | | |
| 2 | 0.96 | 0.62 - 1.47 | 0.842 | 1.02 | 0.67 - 1.57 | 0.916 |
| 3 | 0.91 | 0.63 - 1.32 | 0.614 | 0.99 | 0.68 - 1.44 | 0.952 |
| 4 | 2.75 | 1.93 - 3.90 | <0.001 | 3.06 | 2.12 - 4.42 | <0.001 |
| **AJCC 8th N stage** | | | 0.090 | / | / | / |
| 0 | Ref | | | | | |
| 1 | 1.09 | 0.75 - 1.60 | 0.650 | | | |
| 2 | 1.15 | 0.79 - 1.68 | 0.456 | | | |
| 3 | 1.59 | 1.04 - 2.44 | 0.032 | | | |
| **EBV DNA (copies/mL)** | | | 0.685 | / | / | / |
| <2000 | Ref | | | | | |
| 2000-20000 | 1.17 | 0.82 - 1.68 | 0.385 | | | |
| >20000 | 1.01 | 0.59 - 1.74 | 0.959 | | | |
| **LDH (U/L)** | | | | / | / | / |
| <240 | Ref | | | | | |
| ≥240 | 1.14 | 0.77 - 1.71 | 0.513 | | | |
| **Treatment modalities** | | | 0.002 | | | 0.002 |
| CCRT | Ref | | | Ref | | |
| IMRT | 1.07 | 0.83 - 1.38 | 0.590 | 1.21 | 0.93 - 1.58 | 0.158 |
| IC+CCRT | 1.01 | 0.72 - 1.42 | 0.950 | 0.97 | 0.69 - 1.36 | 0.870 |

| | | | | | | |
|---|---|---|---|---|---|---|
| IC+IMRT | 2.47 | 1.53 - 3.98 | <0.001 | 2.32 | 1.44 - 3.75 | 0.001 |
| CCRT+AC | 0.56 | 0.18 - 1.74 | 0.314 | 0.52 | 0.16 - 1.62 | 0.256 |
| IC+CCRT+AC | 1.21 | 0.38 - 3.82 | 0.745 | 1.30 | 0.41 - 4.09 | 0.659 |
| IMRT+AC | 4.89 | 1.56 - 15.35 | 0.007 | 5.27 | 1.67 - 16.64 | 0.005 |
| IC+IMRT+AC | 0.00 | 0.00 - 4.59E+79 | 0.931 | 0.00 | 0.00 - 1.90E+113 | 0.946 |

Abbreviations: AC, adjuvant chemotherapy; AJCC, American Joint Committee on Cancer; CCRT, concurrent chemoradiotherapy; CI, confidence interval; EBV DNA, Epstein-Barr Virus DNA; IC, induction chemotherapy; IMRT, intensity-modulated radiation therapy; LDH, lactate dehydrogenase; OS, overall survival; Ref, reference; SMR, standardized mortality ratio.

## Supplementary Figures

### Figure S1

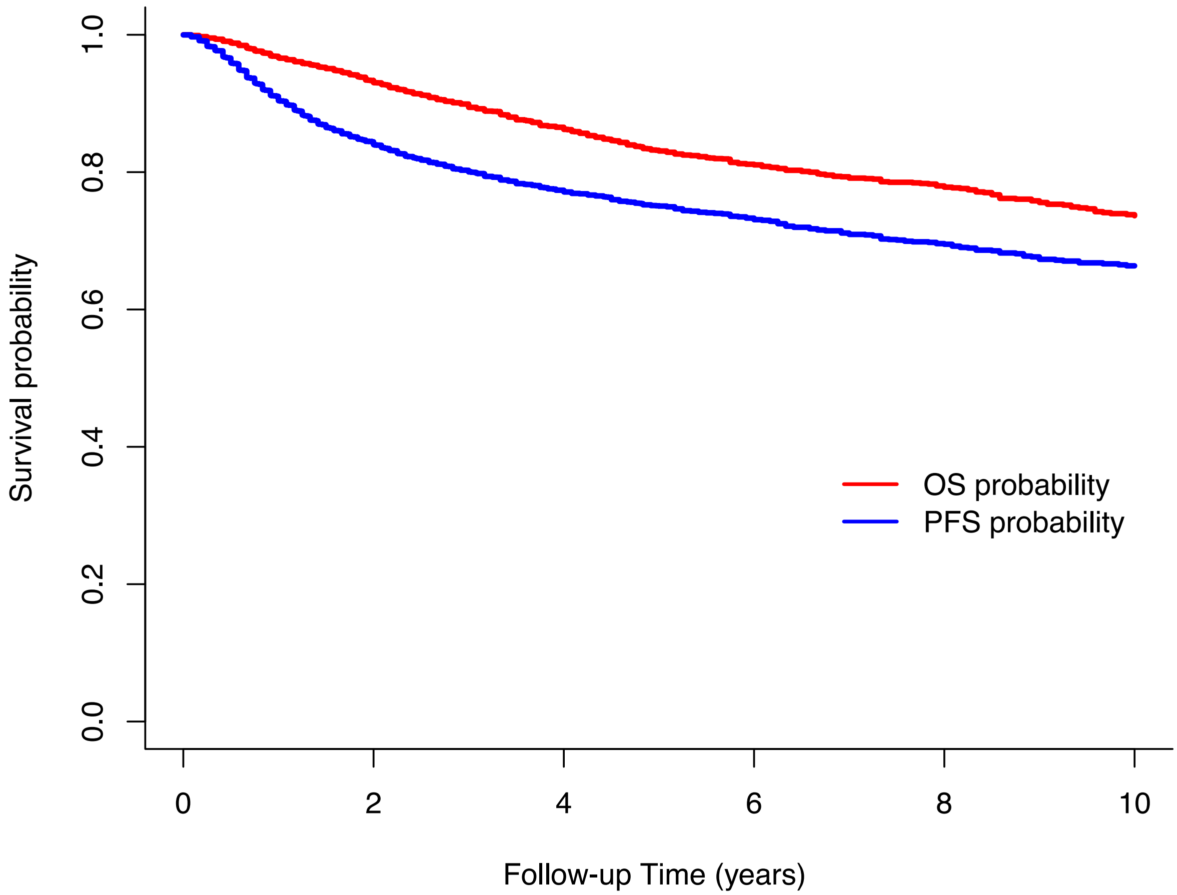


**Figure S1. Survival curves for OS and PFS in the entire cohort.** OS, overall survival; PFS, progression-free survival.

Figure S2

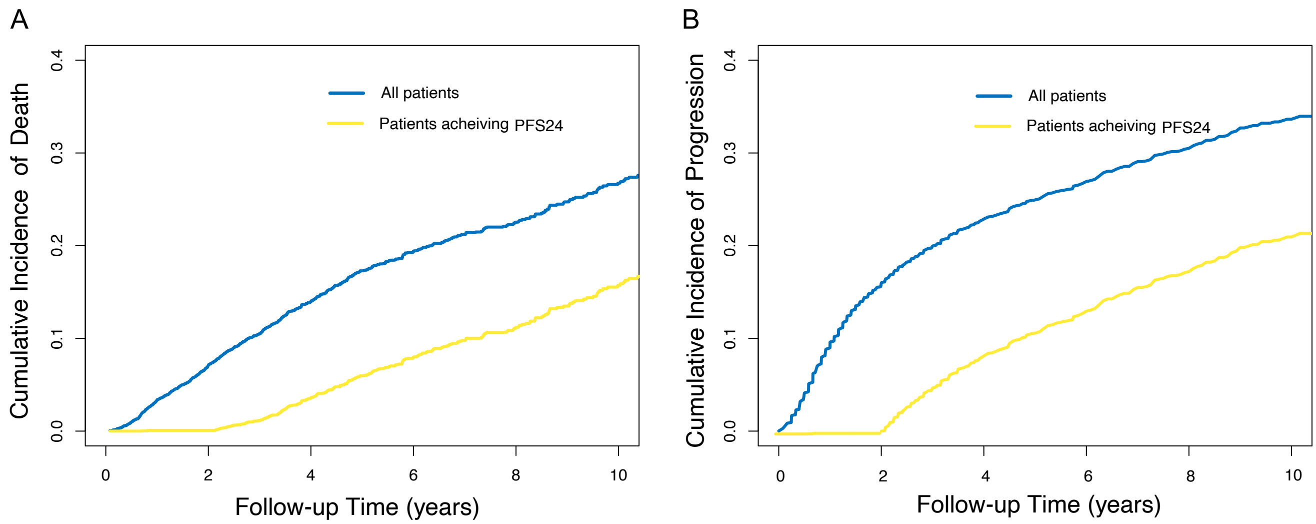


**Figure S2. Event-specific cumulative incidences for NPC patients in the entire cohort compared with patients achieving PFS24.** (A) Cumulative risk of death and (B) cumulative risk of progression. NPC, nasopharyngeal carcinoma; PFS24, 24-month progression-free survival following initial treatment.
